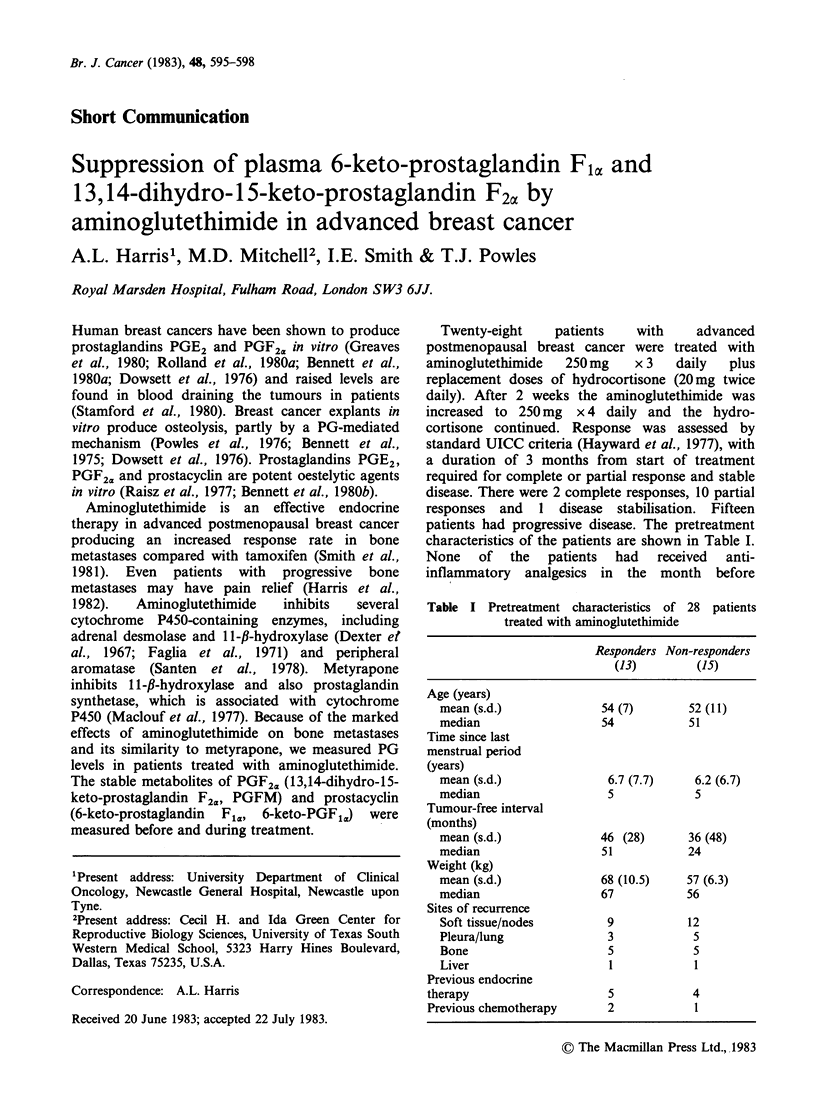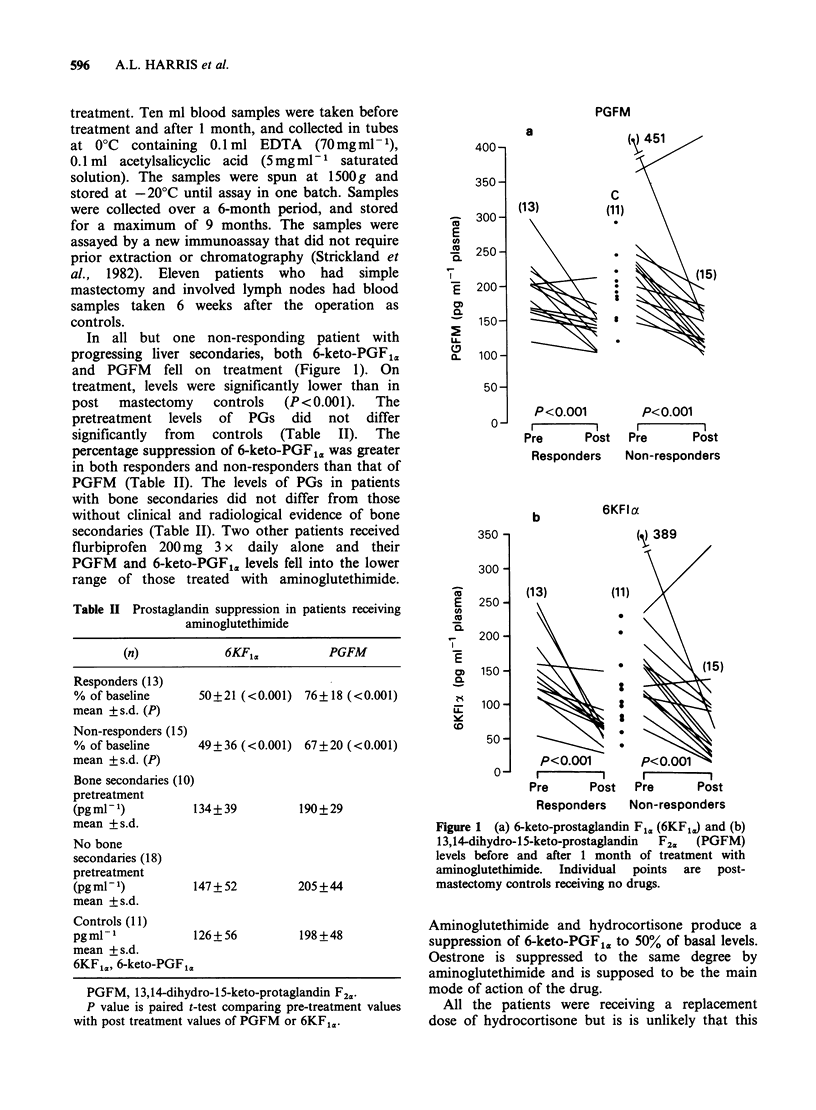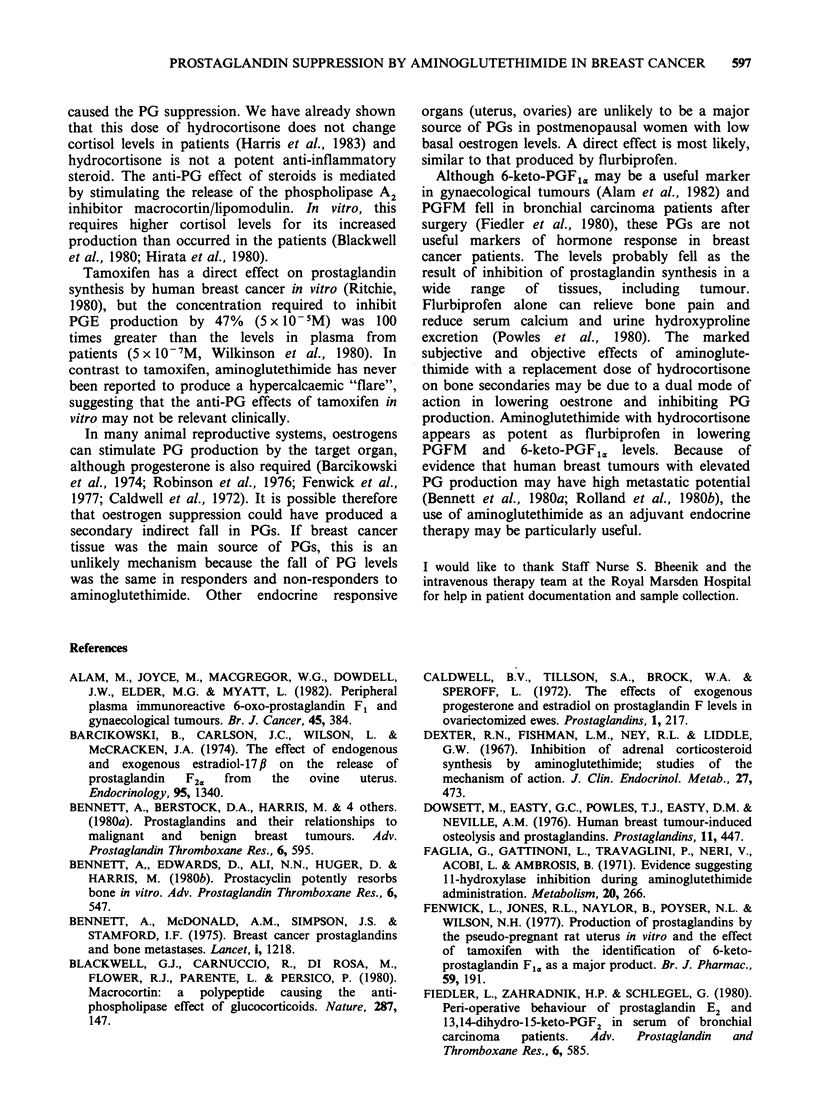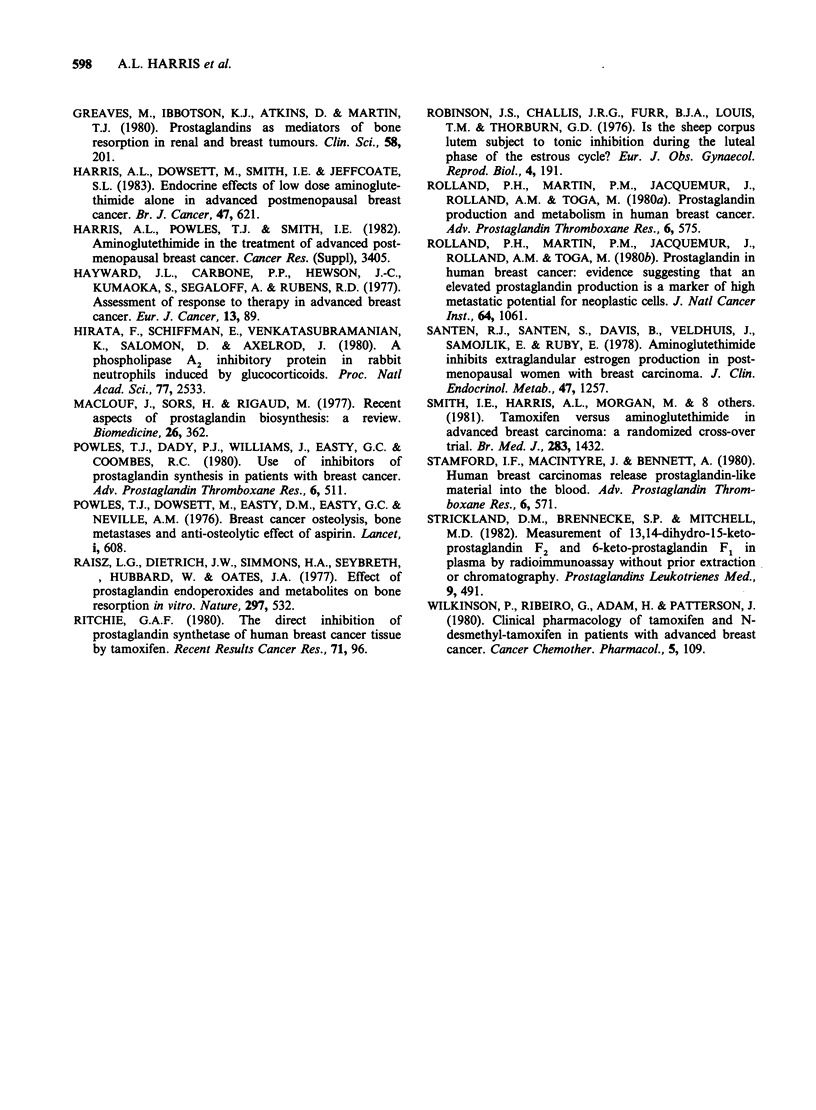# Suppression of plasma 6-keto-prostaglandin F1 alpha and 13,14-dihydro-15-keto-prostaglandin F2 alpha by aminoglutethimide in advanced breast cancer.

**DOI:** 10.1038/bjc.1983.233

**Published:** 1983-10

**Authors:** A. L. Harris, M. D. Mitchell, I. E. Smith, T. J. Powles


*Br. J. Cancer* (1983), **48**, 595–598

### Short Communication

### Suppression of plasma 6-keto-prostaglandin $F_{1\alpha}$ and 13,14-dihydro-15-keto-prostaglandin $F_{2\alpha}$ by aminoglutethimide in advanced breast cancer

A.L. Harris[1], M.D. Mitchell[2], I.E. Smith & T.J. Powles

*Royal Marsden Hospital, Fulham Road, London SW3 6JJ.*

Human breast cancers have been shown to produce prostaglandins $PGE_2$ and $PGF_{2\alpha}$ *in vitro* (Greaves *et al.*, 1980; Rolland *et al.*, 1980*a*; Bennett *et al.*, 1980*a*; Dowsett *et al.*, 1976) and raised levels are found in blood draining the tumours in patients (Stamford *et al.*, 1980). Breast cancer explants *in vitro* produce osteolysis, partly by a PG-mediated mechanism (Powles *et al.*, 1976; Bennett *et al.*, 1975; Dowsett *et al.*, 1976). Prostaglandins $PGE_2$, $PGF_{2\alpha}$ and prostacyclin are potent oestelytic agents *in vitro* (Raisz *et al.*, 1977; Bennett *et al.*, 1980*b*).

Aminoglutethimide is an effective endocrine therapy in advanced postmenopausal breast cancer producing an increased response rate in bone metastases compared with tamoxifen (Smith *et al.*, 1981). Even patients with progressive bone metastases may have pain relief (Harris *et al.*, 1982). Aminoglutethimide inhibits several cytochrome P450-containing enzymes, including adrenal desmolase and 11-$\beta$-hydroxylase (Dexter *et al.*, 1967; Faglia *et al.*, 1971) and peripheral aromatase (Santen *et al.*, 1978). Metyrapone inhibits 11-$\beta$-hydroxylase and also prostaglandin synthetase, which is associated with cytochrome P450 (Maclouf *et al.*, 1977). Because of the marked effects of aminoglutethimide on bone metastases and its similarity to metyrapone, we measured PG levels in patients treated with aminoglutethimide. The stable metabolites of $PGF_{2\alpha}$ (13,14-dihydro-15-keto-prostaglandin $F_{2\alpha}$, PGFM) and prostacyclin (6-keto-prostaglandin $F_{1\alpha}$, 6-keto-$PGF_{1\alpha}$) were measured before and during treatment.

Twenty-eight patients with advanced postmenopausal breast cancer were treated with aminoglutethimide 250 mg ×3 daily plus replacement doses of hydrocortisone (20 mg twice daily). After 2 weeks the aminoglutethimide was increased to 250 mg ×4 daily and the hydrocortisone continued. Response was assessed by standard UICC criteria (Hayward *et al.*, 1977), with a duration of 3 months from start of treatment required for complete or partial response and stable disease. There were 2 complete responses, 10 partial responses and 1 disease stabilisation. Fifteen patients had progressive disease. The pretreatment characteristics of the patients are shown in Table I. None of the patients had received anti-inflammatory analgesics in the month before

**Table I** Pretreatment characteristics of 28 patients treated with aminoglutethimide

| | *Responders (13)* | *Non-responders (15)* |
|---|---|---|
| Age (years) | | |
| mean (s.d.) | 54 (7) | 52 (11) |
| median | 54 | 51 |
| Time since last menstrual period (years) | | |
| mean (s.d.) | 6.7 (7.7) | 6.2 (6.7) |
| median | 5 | 5 |
| Tumour-free interval (months) | | |
| mean (s.d.) | 46 (28) | 36 (48) |
| median | 51 | 24 |
| Weight (kg) | | |
| mean (s.d.) | 68 (10.5) | 57 (6.3) |
| median | 67 | 56 |
| Sites of recurrence | | |
| Soft tissue/nodes | 9 | 12 |
| Pleura/lung | 3 | 5 |
| Bone | 5 | 5 |
| Liver | 1 | 1 |
| Previous endocrine therapy | 5 | 4 |
| Previous chemotherapy | 2 | 1 |

[1]Present address: University Department of Clinical Oncology, Newcastle General Hospital, Newcastle upon Tyne.
[2]Present address: Cecil H. and Ida Green Center for Reproductive Biology Sciences, University of Texas South Western Medical School, 5323 Harry Hines Boulevard, Dallas, Texas 75235, U.S.A.

Correspondence: A.L. Harris

Received 20 June 1983; accepted 22 July 1983.

© The Macmillan Press Ltd., 1983

treatment. Ten ml blood samples were taken before treatment and after 1 month, and collected in tubes at 0°C containing 0.1 ml EDTA (70 mg $ml^{-1}$), 0.1 ml acetylsalicyclic acid (5 mg $ml^{-1}$ saturated solution). The samples were spun at 1500 *g* and stored at −20°C until assay in one batch. Samples were collected over a 6-month period, and stored for a maximum of 9 months. The samples were assayed by a new immunoassay that did not require prior extraction or chromatography (Strickland *et al.*, 1982). Eleven patients who had simple mastectomy and involved lymph nodes had blood samples taken 6 weeks after the operation as controls.

In all but one non-responding patient with progressing liver secondaries, both 6-keto-$PGF_{1\alpha}$ and PGFM fell on treatment (Figure 1). On treatment, levels were significantly lower than in post mastectomy controls ($P<0.001$). The pretreatment levels of PGs did not differ significantly from controls (Table II). The percentage suppression of 6-keto-$PGF_{1\alpha}$ was greater in both responders and non-responders than that of PGFM (Table II). The levels of PGs in patients with bone secondaries did not differ from those without clinical and radiological evidence of bone secondaries (Table II). Two other patients received flurbiprofen 200 mg 3× daily alone and their PGFM and 6-keto-$PGF_{1\alpha}$ levels fell into the lower range of those treated with aminoglutethimide.

**Table II** Prostaglandin suppression in patients receiving aminoglutethimide

| (*n*) | $6KF_{1\alpha}$ | *PGFM* |
|---|---|---|
| Responders (13) % of baseline mean ±s.d. (*P*) | 50±21 (<0.001) | 76±18 (<0.001) |
| Non-responders (15) % of baseline mean ±s.d. (*P*) | 49±36 (<0.001) | 67±20 (<0.001) |
| Bone secondaries (10) pretreatment (pg $ml^{-1}$) mean ±s.d. | 134±39 | 190±29 |
| No bone secondaries (18) pretreatment (pg $ml^{-1}$) mean ±s.d. | 147±52 | 205±44 |
| Controls (11) pg $ml^{-1}$ mean ±s.d. | 126±56 | 198±48 |
| $6KF_{1\alpha}$, 6-keto-$PGF_{1\alpha}$ | | |

PGFM, 13,14-dihydro-15-keto-protaglandin $F_{2\alpha}$.
*P* value is paired *t*-test comparing pre-treatment values with post treatment values of PGFM or $6KF_{1\alpha}$.

**Figure 1** (a) 6-keto-prostaglandin $F_{1\alpha}$ ($6KF_{1\alpha}$) and (b) 13,14-dihydro-15-keto-prostaglandin $F_{2\alpha}$ (PGFM) levels before and after 1 month of treatment with aminoglutethimide. Individual points are post-mastectomy controls receiving no drugs.

Aminoglutethimide and hydrocortisone produce a suppression of 6-keto-$PGF_{1\alpha}$ to 50% of basal levels. Oestrone is suppressed to the same degree by aminoglutethimide and is supposed to be the main mode of action of the drug.

All the patients were receiving a replacement dose of hydrocortisone but is is unlikely that this caused the PG suppression. We have already shown that this dose of hydrocortisone does not change cortisol levels in patients (Harris *et al.*, 1983) and hydrocortisone is not a potent anti-inflammatory steroid. The anti-PG effect of steroids is mediated by stimulating the release of the phospholipase $A_2$ inhibitor macrocortin/lipomodulin. *In vitro,* this requires higher cortisol levels for its increased production than occurred in the patients (Blackwell *et al.*, 1980; Hirata *et al.*, 1980).

Tamoxifen has a direct effect on prostaglandin synthesis by human breast cancer *in vitro* (Ritchie, 1980), but the concentration required to inhibit PGE production by 47% ($5 \times 10^{-5}$M) was 100 times greater than the levels in plasma from patients ($5 \times 10^{-7}$M, Wilkinson *et al.*, 1980). In contrast to tamoxifen, aminoglutethimide has never been reported to produce a hypercalcaemic "flare", suggesting that the anti-PG effects of tamoxifen *in vitro* may not be relevant clinically.

In many animal reproductive systems, oestrogens can stimulate PG production by the target organ, although progesterone is also required (Barcikowski *et al.*, 1974; Robinson *et al.*, 1976; Fenwick *et al.*, 1977; Caldwell *et al.*, 1972). It is possible therefore that oestrogen suppression could have produced a secondary indirect fall in PGs. If breast cancer tissue was the main source of PGs, this is an unlikely mechanism because the fall of PG levels was the same in responders and non-responders to aminoglutethimide. Other endocrine responsive organs (uterus, ovaries) are unlikely to be a major source of PGs in postmenopausal women with low basal oestrogen levels. A direct effect is most likely, similar to that produced by flurbiprofen.

Although 6-keto-$PGF_{1\alpha}$ may be a useful marker in gynaecological tumours (Alam *et al.*, 1982) and PGFM fell in bronchial carcinoma patients after surgery (Fiedler *et al.*, 1980), these PGs are not useful markers of hormone response in breast cancer patients. The levels probably fell as the result of inhibition of prostaglandin synthesis in a wide range of tissues, including tumour. Flurbiprofen alone can relieve bone pain and reduce serum calcium and urine hydroxyproline excretion (Powles *et al.*, 1980). The marked subjective and objective effects of aminoglutethimide with a replacement dose of hydrocortisone on bone secondaries may be due to a dual mode of action in lowering oestrone and inhibiting PG production. Aminoglutethimide with hydrocortisone appears as potent as flurbiprofen in lowering PGFM and 6-keto-$PGF_{1\alpha}$ levels. Because of evidence that human breast tumours with elevated PG production may have high metastatic potential (Bennett *et al.*, 1980*a*; Rolland *et al.*, 1980*b*), the use of aminoglutethimide as an adjuvant endocrine therapy may be particularly useful.

I would like to thank Staff Nurse S. Bheenik and the intravenous therapy team at the Royal Marsden Hospital for help in patient documentation and sample collection.

### References

ALAM, M., JOYCE, M., MACGREGOR, W.G., DOWDELL, J.W., ELDER, M.G. & MYATT, L. (1982). Peripheral plasma immunoreactive 6-oxo-prostaglandin $F_1$ and gynaecological tumours. *Br. J. Cancer,* **45,** 384.

BARCIKOWSKI, B., CARLSON, J.C., WILSON, L. & McCRACKEN, J.A. (1974). The effect of endogenous and exogenous estradiol-17$\beta$ on the release of prostaglandin $F_{2\alpha}$ from the ovine uterus. *Endocrinology,* **95,** 1340.

BENNETT, A., BERSTOCK, D.A., HARRIS, M. & 4 others. (1980*a*). Prostaglandins and their relationships to malignant and benign breast tumours. *Adv. Prostaglandin Thromboxane Res.,* **6,** 595.

BENNETT, A., EDWARDS, D., ALI, N.N., HUGER, D. & HARRIS, M. (1980*b*). Prostacyclin potently resorbs bone *in vitro. Adv. Prostaglandin Thromboxane Res.,* **6,** 547.

BENNETT, A., McDONALD, A.M., SIMPSON, J.S. & STAMFORD, I.F. (1975). Breast cancer prostaglandins and bone metastases. *Lancet,* **i,** 1218.

BLACKWELL, G.J., CARNUCCIO, R., DI ROSA, M., FLOWER, R.J., PARENTE, L. & PERSICO, P. (1980). Macrocortin: a polypeptide causing the anti-phospholipase effect of glucocorticoids. *Nature,* **287,** 147.

CALDWELL, B.V., TILLSON, S.A., BROCK, W.A. & SPEROFF, L. (1972). The effects of exogenous progesterone and estradiol on prostaglandin F levels in ovariectomized ewes. *Prostaglandins,* **1,** 217.

DEXTER, R.N., FISHMAN, L.M., NEY, R.L. & LIDDLE, G.W. (1967). Inhibition of adrenal corticosteroid synthesis by aminoglutethimide; studies of the mechanism of action. *J. Clin. Endocrinol. Metab.,* **27,** 473.

DOWSETT, M., EASTY, G.C., POWLES, T.J., EASTY, D.M. & NEVILLE, A.M. (1976). Human breast tumour-induced osteolysis and prostaglandins. *Prostaglandins,* **11,** 447.

FAGLIA, G., GATTINONI, L., TRAVAGLINI, P., NERI, V., ACOBI, L. & AMBROSIS, B. (1971). Evidence suggesting 11-hydroxylase inhibition during aminoglutethimide administration. *Metabolism,* **20,** 266.

FENWICK, L., JONES, R.L., NAYLOR, B., POYSER, N.L. & WILSON, N.H. (1977). Production of prostaglandins by the pseudo-pregnant rat uterus *in vitro* and the effect of tamoxifen with the identification of 6-keto-prostaglandin $F_{1\alpha}$ as a major product. *Br. J. Pharmac.,* **59,** 191.

FIEDLER, L., ZAHRADNIK, H.P. & SCHLEGEL, G. (1980). Peri-operative behaviour of prostaglandin $E_2$ and 13,14-dihydro-15-keto-$PGF_2$ in serum of bronchial carcinoma patients. *Adv. Prostaglandin and Thromboxane Res.,* **6,** 585.

GREAVES, M., IBBOTSON, K.J., ATKINS, D. & MARTIN, T.J. (1980). Prostaglandins as mediators of bone resorption in renal and breast tumours. *Clin. Sci.*, **58**, 201.

HARRIS, A.L., DOWSETT, M., SMITH, I.E. & JEFFCOATE, S.L. (1983). Endocrine effects of low dose aminoglutethimide alone in advanced postmenopausal breast cancer. *Br. J. Cancer*, **47**, 621.

HARRIS, A.L., POWLES, T.J. & SMITH, I.E. (1982). Aminoglutethimide in the treatment of advanced postmenopausal breast cancer. *Cancer Res.* (Suppl), 3405.

HAYWARD, J.L., CARBONE, P.P., HEWSON, J.-C., KUMAOKA, S., SEGALOFF, A. & RUBENS, R.D. (1977). Assessment of response to therapy in advanced breast cancer. *Eur. J. Cancer*, **13**, 89.

HIRATA, F., SCHIFFMAN, E., VENKATASUBRAMANIAN, K., SALOMON, D. & AXELROD, J. (1980). A phospholipase $A_2$ inhibitory protein in rabbit neutrophils induced by glucocorticoids. *Proc. Natl Acad. Sci.*, **77**, 2533.

MACLOUF, J., SORS, H. & RIGAUD, M. (1977). Recent aspects of prostaglandin biosynthesis: a review. *Biomedicine*, **26**, 362.

POWLES, T.J., DADY, P.J., WILLIAMS, J., EASTY, G.C. & COOMBES, R.C. (1980). Use of inhibitors of prostaglandin synthesis in patients with breast cancer. *Adv. Prostaglandin Thromboxane Res.*, **6**, 511.

POWLES, T.J., DOWSETT, M., EASTY, D.M., EASTY, G.C. & NEVILLE, A.M. (1976). Breast cancer osteolysis, bone metastases and anti-osteolytic effect of aspirin. *Lancet*, **i**, 608.

RAISZ, L.G., DIETRICH, J.W., SIMMONS, H.A., SEYBRETH, , HUBBARD, W. & OATES, J.A. (1977). Effect of prostaglandin endoperoxides and metabolites on bone resorption *in vitro*. *Nature*, **297**, 532.

RITCHIE, G.A.F. (1980). The direct inhibition of prostaglandin synthetase of human breast cancer tissue by tamoxifen. *Recent Results Cancer Res.*, **71**, 96.

ROBINSON, J.S., CHALLIS, J.R.G., FURR, B.J.A., LOUIS, T.M. & THORBURN, G.D. (1976). Is the sheep corpus lutem subject to tonic inhibition during the luteal phase of the estrous cycle? *Eur. J. Obs. Gynaecol. Reprod. Biol.*, **4**, 191.

ROLLAND, P.H., MARTIN, P.M., JACQUEMUR, J., ROLLAND, A.M. & TOGA, M. (1980*a*). Prostaglandin production and metabolism in human breast cancer. *Adv. Prostaglandin Thromboxane Res.*, **6**, 575.

ROLLAND, P.H., MARTIN, P.M., JACQUEMUR, J., ROLLAND, A.M. & TOGA, M. (1980*b*). Prostaglandin in human breast cancer: evidence suggesting that an elevated prostaglandin production is a marker of high metastatic potential for neoplastic cells. *J. Natl Cancer Inst.*, **64**, 1061.

SANTEN, R.J., SANTEN, S., DAVIS, B., VELDHUIS, J., SAMOJLIK, E. & RUBY, E. (1978). Aminoglutethimide inhibits extraglandular estrogen production in postmenopausal women with breast carcinoma. *J. Clin. Endocrinol. Metab.*, **47**, 1257.

SMITH, I.E., HARRIS, A.L., MORGAN, M. & 8 others. (1981). Tamoxifen versus aminoglutethimide in advanced breast carcinoma: a randomized cross-over trial. *Br. Med. J.*, **283**, 1432.

STAMFORD, I.F., MACINTYRE, J. & BENNETT, A. (1980). Human breast carcinomas release prostaglandin-like material into the blood. *Adv. Prostaglandin Thromboxane Res.*, **6**, 571.

STRICKLAND, D.M., BRENNECKE, S.P. & MITCHELL, M.D. (1982). Measurement of 13,14-dihydro-15-keto-prostaglandin $F_2$ and 6-keto-prostaglandin $F_1$ in plasma by radioimmunoassay without prior extraction or chromatography. *Prostaglandins Leukotrienes Med.*, **9**, 491.

WILKINSON, P., RIBEIRO, G., ADAM, H. & PATTERSON, J. (1980). Clinical pharmacology of tamoxifen and N-desmethyl-tamoxifen in patients with advanced breast cancer. *Cancer Chemother. Pharmacol.*, **5**, 109.